# Selective Adaptation in Networks of Heterogeneous Populations: Model, Simulation, and Experiment

**DOI:** 10.1371/journal.pcbi.0040029

**Published:** 2008-02-15

**Authors:** Avner Wallach, Danny Eytan, Shimon Marom, Ron Meir

**Affiliations:** 1 Faculty of Electrical Engineering, Technion Israel Institute of Technology, Haifa, Israel; 2 Faculty of Medicine, Technion Israel Institute of Technology, Haifa, Israel; University College London, United Kingdom

## Abstract

Biological systems often change their responsiveness when subject to persistent stimulation, a phenomenon termed adaptation. In neural systems, this process is often selective, allowing the system to adapt to one stimulus while preserving its sensitivity to another. In some studies, it has been shown that adaptation to a frequent stimulus increases the system's sensitivity to rare stimuli. These phenomena were explained in previous work as a result of complex interactions between the various subpopulations of the network. A formal description and analysis of neuronal systems, however, is hindered by the network's heterogeneity and by the multitude of processes taking place at different time-scales. Viewing neural networks as populations of interacting elements, we develop a framework that facilitates a formal analysis of complex, structured, heterogeneous networks. The formulation developed is based on an analysis of the availability of activity dependent resources, and their effects on network responsiveness. This approach offers a simple mechanistic explanation for selective adaptation, and leads to several predictions that were corroborated in both computer simulations and in cultures of cortical neurons developing in vitro. The framework is sufficiently general to apply to different biological systems, and was demonstrated in two different cases.

## Author Summary

Our mind continuously adapts to background sensory events while preserving and even enhancing its sensitivity to deviant objects. In the visual modality, for instance, a target violating a surrounding pattern is easily detected, a phenomenon termed “pop-out.” Indeed, the automatic attention we pay to the irregular or the surprising, which developed as a valuable aid in our survival, is often used to advantage nowadays in popular culture and advertisement. Such phenomena have been investigated in many systems, from psychophysics in behaving animals to experiments in neural networks developing in vitro. In this work, we develop a mechanistic model that demonstrates how a relatively simple system may express such selective behavior. We apply our model first to the case of transiently responding networks, and compare the results with computer simulations and experimental data collected from neural networks developing in vitro. We also demonstrate the application of the model to other systems. Our approach provides insight as to how complex, behavior-related phenomena may arise from simple dynamic interactions between the system's elementary components.

## Introduction

Adaptation is a biologically ubiquitous process whereby features of a system's responsiveness change as a result of previous input. In neural systems, the kinetics of the change are most often monotonic and its direction (either increase or decrease) depends on the information conveyed and on the identity of the biological components observed (e.g., [1–3]). It has been shown that neural adaptation is inherently selective to the input characteristics; not only between sensory modalities, but even within a given modality, the system is capable of reducing its sensitivity to frequent input while preserving its sensitivity to the rare (e.g., [3–8]). In its most impressive form, the phenomenon of selective adaptation also contains an increased sensitivity to the rare on the background of frequent input. For instance, in the phenomenon of Mismatch Negativity (MMN), when a deviant sensory stimulus is applied on the background of a standard one, an evoked potential component which is absent in the presence of a single stimulus (e.g., [6,9,10]) is generated. This component's magnitude was shown to depend on the scarcity of the odd-ball stimulus and on the rate of stimulation.

To simplify matters, adaptation can be viewed as a result of a dynamic interaction between exciting and restoring forces: the monotonic nature of adaptation, be it facilitation or depression, is explained in terms of net loss or gain of exciting or restoring resources. For instance, one mechanism for the monotonic decrease in excitability in a cortical neuron under repetitive stimulation was shown to be an increase in effective potassium membrane conductance [11]. The inverse effect, that is increased excitability, can result from, e.g., the cumulative inactivation of potassium membrane conductance [12]. Such an interplay between opponent processes abounds in models of adaptation.

The selective nature of adaptation is usually interpreted in terms of the spatial locality of the above processes. For instance, [13] showed that constraining the monotonic decrease to a unique subset of synapses allows for selective adaptation of a single neuron to the wide range of inputs received.

A neural correlate of increased sensitivity to the rare, a phenomenon most commonly documented in auditory event related potentials [6], was observed at the level of random networks of cortical neurons in vitro[14,15], where the mechanism underlying this complex phenomenon is accessible. Using a combination of multi-site recordings, and pharmacological and electrophysiological manipulations Eytan at al. suggested the following possible mechanism (see schematic illustration in Figure 1): It has been shown in numerous studies that cortical inhibitory interneurons form extended, gap-junction coupled sub-networks [16]. We can hypothesize that such expansive electrically coupled networks are relatively insensitive to the site of stimulation, thus acting as a global component of the system. In contrast, the excitatory population is uncoupled electrically (e.g., [17]) and therefore its activation is pathway specific and is affected locally by stimulation. When one site in the network is stimulated frequently, the local excitatory pathways stemming from this source undergo depression which causes a decline in the network's responsiveness to this stimulation. The inhibitory sub-network is also affected by the frequent stimulation, *albeit globally*, and undergoes depression. The net result is less resistance to the activation pathways stemming from other stimulation sites, hence the enhanced responsiveness to other, less frequently stimulated sites. To support this explanation, Eytan et al. demonstrated that blocking the inhibitory sub-network (by using the GABA antagonist Bicuculline) abolished the increase in the sensitivity to rare stimulation.

**Figure 1:**
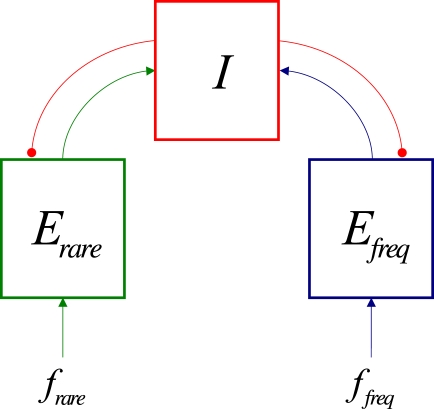
Diagram of Dual Site Stimulation Model Stimulation effect on the excitatory populations is local, and therefore there is stronger depression of that population at the frequent site. The effect on the inhibitory resource, however, is global due to its interconnectivity so it is affected by stimulation at all sites. Thus, response to the rare site may be increased due to the global decline in inhibition.

In what follows, we formulate a generic mathematical model for the full range of adaptation phenomena, that is: (*i)* Monotonic facilitation or depression of responsiveness; (*ii*) Selectivity of adaptation, and *(iii)* Increased sensitivity to rare stimuli. The model consists of two nonlinear differential equations, describing the effect of stimulation on the system's exciting and restoring resources. These equations are coupled via functions describing the activities of the two components of the system. This approach enables us to reduce the spatial structure of the system (i.e., globality versus locality of its different components) into simple, input-output relationships.

To demonstrate its generic nature, the model was applied to two distinctly different systems. First, we develop the equations for transiently responding neural networks. The behavior of such networks is characterized by low tonic activity with reverberating transient responses to stimulation. After formalizing and analyzing this model, we proceed by verifying it using computer simulations and multi-site recordings of large random networks of cortical neurons developing in vitro. Second, we analyze a generic system of coupled populations that maintains a high level of tonic activity in response to steady external stimulation.

Our main contributions in this work are the development of a methodology that facilitates a formal analysis of structurally complex and heterogeneous networks, and the implementation of this methodology to investigate adaptation processes in neural systems. We expect this framework to be applicable to other biological systems as well.

## Results

### Activity-Dependent Resources Model

We consider a system consisting of two components, one excitatory and one inhibitory. The availability of these components is use-dependent, so that the activity of each component results in a decline in its resources. The replenishment process of each resource is assumed to follow first order dynamics with a characteristic time constant. Formally, this can be written

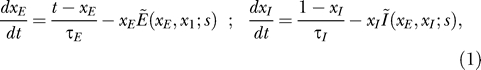
where *x*_E_, *x*_I_ are the relative availabilities of each resource, *t*_E,_ *t*_I_ are the characteristic time constants and 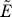 and 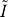 are the activity in each component of the system. We assume here that the activity depends on the state of the system (i.e., resource availabilities) and on external stimulation.

Now let us assume that the activity in each component has a multiplicative dependence on stimulation intensity, which is a function of one or more parameters of the stimulus (e.g., amplitude, rate, density etc.), namely

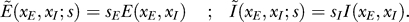



Here, *s*_E_, *s*_I_ are the effective intensities of stimulation of each component. By this we assume that each component may be affected differently by each stimulation source, or that it may be affected by a different number of sources. Equation 1 can therefore be rewritten

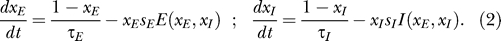



We define the system's responsiveness as the excitatory activity, normalized to its non-adapted value

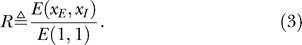



By this we assume that most activity in experimental recordings results from the excitatory cells. Since Equation 3 does not depend explicitly on input intensity (see the examples below), the responsiveness can be viewed as the site's normalized input-output gain.

At this stage we do not specify the exact activity functions *E* and *I*. However, we can make several reasonable assumptions:

Inhibitory activity is explicitly dependent on excitatory activity:

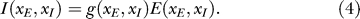



This non-restrictive assumption holds whenever the inhibitory population is not driven directly by external stimulation (see for example the tonic system discussed below).

Increasing the excitatory synaptic availability, *x*_E_, increases the excitatory activity, i.e., the responsiveness is a monotonically non-decreasing function of *x*_E_,

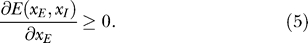



Increasing the inhibitory synaptic availability, *x*_I_, decreases the excitatory activity, i.e., the responsiveness is a monotonically non-increasing function of *x*_I_,

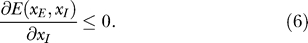



The assumptions in Equations 5 and 6 refer only to the excitatory activity due to our definition of the system's responsiveness in Equation 3.

In what follows we analyze the system in Equation 2, using the above assumptions, in two qualitatively different instances. First, we consider the case of a network with transient responses, to which a periodic stimulation is applied. The results are then compared with experiments in neural networks developing in vitro. Second, we analyze a general network with tonic activity, to which a constant stimulus is applied.

### Model Network with Transient Response

The detailed derivation of Equation 2 for a transiently responding network, which we used as a reference case, is described in Materials and Methods. In that particular application the stimulation is periodic with an intensity that depends on its period. Thus, Equation 2 for such systems can written

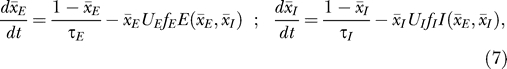
where 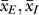 are the time-averaged synaptic availabilities, *U*_E_, *U*_I_ the synaptic vesicle release probabilities and *f*_E_=1/*T*_E_ and *f*_I_=1/*T*_I_ are the stimulation rates (see Materials and Methods for a derivation, including an explanation for the appearance of the temporal averages). If there are several sources of stimulation in the system, *f*_E_ and *f*_I_ can express the total or *effective* rate of stimulation influencing each population.

The system described by Equation 2 converges to fixed points in the 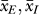 plane (referred to as the phase plane), i.e., the loci at which both derivatives vanish. For simplicity, we assume throughout the analysis of this system a linear relationship between the activity functions

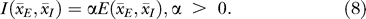



In this case, all the fixed points are located along the curve

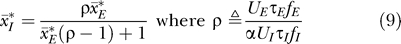
(see Materials and Methods) as illustrated in Figure 2. The parameter ρ expresses the ratio between the net effect of stimulation on the excitatory population to its effect on the inhibitory population; e.g., if ρ is larger than 1, the excitatory population will suffer more resource depletion than the inhibitory one. Alternatively, when ρ < 1, the fixed points are all below the diagonal of the phase plane, where inhibition is more depressed than excitation. Thus, ρ determines the geometric curve upon which the fixed point is located. The exact location of the fixed point along these curves is determined by the specific parameter values and activity functions.

**Figure 2:**
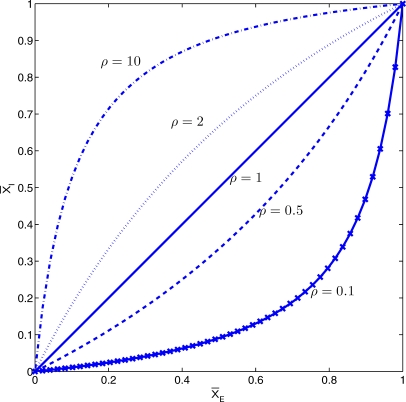
Fixed Point Loci When the Activities are Proportional Geometrical loci of the averaged system's fixed points when the excitatory and inhibitory activity function are proportional to each other, as described in Equation 8. The locus depends on the parameter ratio *ρ*. When *ρ* < 1 fixed points are located in the lower half of the plane (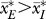 ) and vice versa. We can infer from the assumptions in Equations 5 and 6 that as fixed points approach the lower-right corner of the phase plane, activity increases. Thus, decreasing *ρ* changes the overall behavior from a depressing to a facilitating one.

### Frequency-Dependent Adaptation in a Transiently Responding Network

It was shown in [14] that periodic stimulation leads to a decline in the response to each stimulus. Furthermore, this adaptation process becomes more pronounced as the stimulation frequency is increased. In our model, this corresponds to

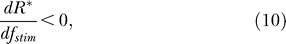
where *f*_stim_ is the stimulation frequency and the asterisk denotes the steady-state value. We will refer to this phenomenon as “frequency dependent adaptation.” Describing this rather trivial phenomenon will serve as a simple case study which will aid us in understanding the model's behavior.

For the purpose of analytic tractability, let us consider a much simplified symmetric model in which

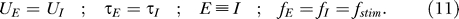



In this case ρ = 1 and all fixed points are on the diagonal 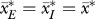 . It is easy to see from Equation 7 that at extremely low stimulation frequency the system converges close to 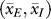 = (1,1) where synaptic availabilities are maximal. As the frequency is increased the fixed point shifts along the diagonal towards the origin 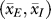 = (0,0) where synaptic depletion is maximal. Therefore, any activity function *E* which monotonically increases on the diagonal will satisfy Equation 10 and exhibit frequency dependent adaptation. Furthermore, such a function also guarantees the fixed point's stability (see Material and Methods, Equation 27). This can be easily understood intuitively since an increasing activity function creates a negative feedback loop between activity and synaptic availability, which results in a stable steady-state solution.

Consider an example of a two-variable sigmoidal function

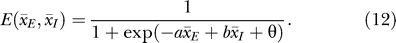



It is easy to verify that if 0 < *b* < *a* then indeed on the diagonal

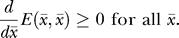



That is to say, when activity depends more strongly on the availability of excitation than on that of inhibition, frequency dependent adaptation will be manifested. This is illustrated in Figure 3.

**Figure 3:**
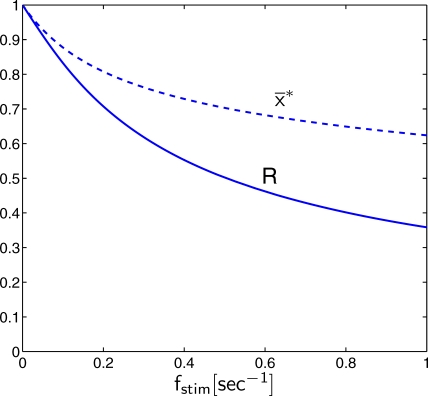
Frequency-Dependent Adaptation in the Theoretical Symmetric System State variables and responsiveness decrease monotonically as stimulation frequency is increased. The curve was calculated for the symmetric system satisfying Equation 11 where the system's activity functions are the sigmoidal activity function given in Equation 12 (*a* = 16.67; *b* = 10; *θ* = 5; *U* = 0.5; *τ* = 10 s).

### Selective Adaptation in Transiently Responding Networks

Next, we consider the two site stimulation protocol studied in [14], where it was demonstrated experimentally that when one site is stimulated frequently and another site is stimulated rarely, the system reduces its response to the frequent stimulus while enhancing its response to the rare one. It was suggested in [14] that the topological differences between the excitatory and inhibitory subnetworks, and specifically the interconnections within the inhibitory population (e.g., [18]) underlie this phenomenon, whereby the inhibitory population acts as a global common resource affected by stimuli from all sites, while the excitatory network is affected mainly by local stimuli.

Under these assumptions the excitatory populations corresponding to the two sites do not influence each other directly and function as two separate components. The inhibitory population, however, is common (see diagram in Figure 1) and therefore responds to **both** stimulation sites. In our model, we can easily realize this mechanism by postulating the existence of two excitatory populations and writing the state equations (Equation 7) for each stimulation site. Thus, the response to the rarely stimulated site will be according to

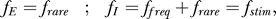
while at the frequent site

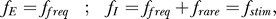
where *f*_stim_ is the total stimulation intensity applied to the system. Thus the parameter ratio *ρ* of Equation 9 is different at each site

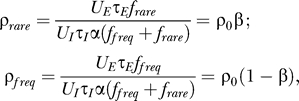
with

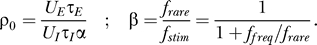



*ρ*_0_ is the stimuli-independent parameter ratio and *β* is the normalized intensity of the rare stimulation site (0 ≤ *β* ≤ 0.5). Since *ρ_rare_* < *ρ_freq_*, the adaptation behavior of the rare site will differ from that of the frequent site.

We now define two functionality criteria for this paradigm. “Selectivity” is defined to be the ratio between the steady-state responsiveness at the two sites,

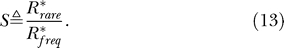



“Amplification” is defined to be the steady-state increase in the system's sensitivity to the rare stimuli,

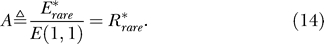



The dependence of *S* and *A* on both the relative intensity *β* and on the total intensity *f*_stim_, for the sigmoidal activity function in Equation 12 is displayed in Figure 4, demonstrating the phenomenon of selective adaptation. The model predicts that selectivity increases with frequency, and that this increase is more pronounced for smaller values of *β*, i.e., when there is a large difference between the stimulation effect on the two subnetworks. The same holds for the amplification criterion, only that for large values of *β* no amplification occurs at all (i.e., *A* < 1).

**Figure 4:**
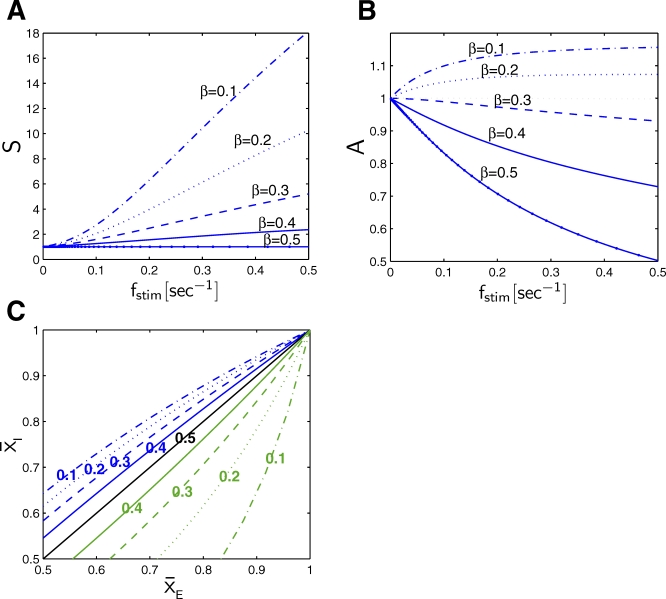
Selective Adaptation in the Theoretical Model (A) Selectivity as defined in Equation 13, for a selective adaptation paradigm using the sigmoidal activity function given in Equation 12 (parameter values as in Figure 3). Here we also assume *ρ*_0_ = 2. Selectivity is monotonically increasing in *f*_stim_ and decreasing in *β*. (B) Amplification as defined in Equation 14, for the same paradigm. For high values of *β* (here, for *β* = 0.5, 0.4, and 0.3) amplification is decreasing with stimulation frequency and is below 1 (i.e., the response to the rare is in fact depressed). When *β* is further decreased (here, for *β* = 0.2 and 0.1), amplification becomes non-decreasing and above 1 so that the response is indeed amplified. (C) phase plane loci of fixed points for the rare (green) and frequent (blue) sites for different values of *β* (marked on the curves). Note that for each value of *β*, *ρ*_0_ = *ρ_rare_* + *ρ_freq_* so that when the locus curve of the rare shifts towards the lower right corner (i.e., towards amplification) the locus curve of the frequent shifts towards the upper left corner (i.e., towards stronger depression).

So far, we have developed an averaged model consisting of a single equation for each synaptic population. Using the linear relationship assumption in Equation 8 we located the fixed point of this system on the geometric curves described in Equation 9. At low stimulation rates, all these curves converge to 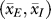 = (1,1). As the rates are increased, the curves diverge and the equilibrium location depends more strongly on the relative intensity *β*. Since the fixed point's location determines the system's responsiveness, assuming integrated activity to be monotonically dependent on resource availability (Equations 5 and 6) leads to the above predictions for dual site stimulation. Note that at high rates of stimulation (∼100 s^−1^) the curves converge towards the origin, where the responsiveness to both sites is extremely low. Since such high stimulation rates are not physiologically possible in in vitro networks, this region does not appear in our analysis. Although we used sigmoidal activity functions for the visualization of these predictions (presented in Figure 4), any activity function obeying Equations 4–6 will exhibit the same behavior.

In summary, the theoretical model we have presented so far explains selective adaptation in terms of the change in the availability of the activity dependent resources of the system. The model predicts that selectivity (i.e., the ability of the system to distinguish between different sources) and amplification (i.e., the system's capability to increase its responsiveness to rare events) both depend on stimulation intensity and on the difference between the stimulation sources.

### Transiently Responding Networks: Simulation Results

A network of leaky integrate-and-fire (LIF) elements was simulated in order to demonstrate the validity of our approach and averaging procedures. It is not meant to provide a comprehensive simulation study of the phenomena. The simulation parameters and setup are detailed in Materials and Methods. Note that all excitatory and inhibitory parameters, as well as the distribution of synapses for all neurons are equal so that the network realizes the symmetrical case in Equation 11. Computing *E* and *I* for various values of 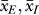 verified that they are indeed similar (data not shown).

Figure 5 depicts the network dynamics for single site stimulation. Panel A shows both excitatory and inhibitory activity during a single transient response. Panel B shows the decrease in normalized network responsiveness towards the steady-state level. Panels C and D illustrate the dynamics of the excitatory and inhibitory depression variables, respectively, and compares their values with those predicted by the averaged equations.

**Figure 5:**
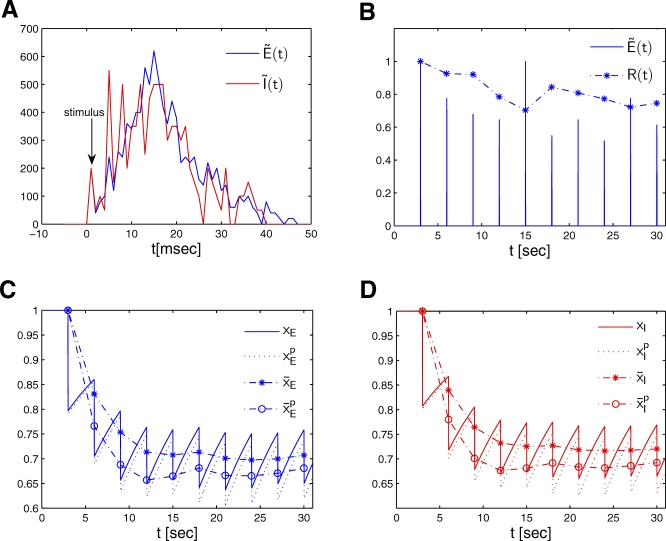
Single Site Stimulation in a Simulated Network The simulation model consisted of a network of leaky integrate and fire neurons with depressing synapses, as described in Materials and Methods. (A) The network is stimulated every 3 s to produce a transient response of both the inhibitory and the excitatory subnetworks. (B) The network adapts to the stimulus and the total responsiveness (as defined in Equation 3) declines. (C, D) Comparing the slow depression variables, averaged over the populations, *x_E_* and *x_I_*, and the value predicted from the network's activity, 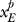 and 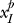 (using Equation 20), where 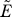 and 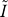 are the activities obtained from the simulation), validates our assumptions for averaging over neural ensembles. Furthermore, the time-averaged variables 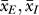 and their predicted value 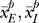 (calculated from the averaged network's activity using Equation 7), where *E* and *I* are the integrated activity obtained from the simulation) converge to a similar steady-state value, which validates our assumptions for averaging over the stimulation period.

Single site stimulation was repeated for various stimulation frequencies, as shown in Figure 6. Average steady-state values of 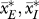 and their predicted values 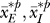 , calculated from the network's activity using Equation 7, were computed so that the averaging procedure is validated for the entire frequency range (A and B). These values, presented in the phase plane, follow closely the fixed-point loci for *ρ* = 1 depicted in Figure 2 (C). The experimental phenomenon of reduced responsiveness is reconstructed in D.

**Figure 6:**
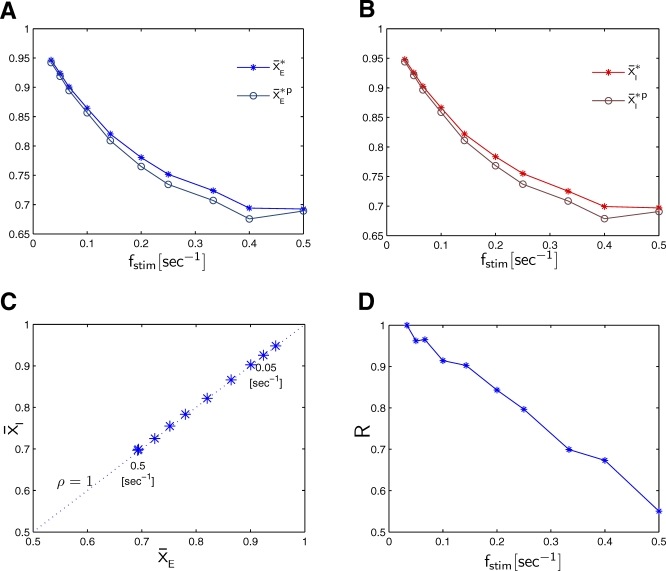
Frequency-Dependent Adaptation in Simulated Networks A total of 10 different networks were stimulated repeatedly at various frequencies (1/30 s^−1^, 1/20 s^−1^, 1/15 s^−1^, 1/10 s^−1^, 1/7 s^−1^, 1/5 s^−1^, 1/4 s^−1^, 1/3 s^−1^, 1/2 s^−1^), and the results were averaged. (A, B) Steady-state values of the state variables 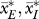 and their predicted values 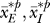 (see Figure 5) were computed from the last 8 cycles of each simulation. (C) The phase plane fixed points are indeed located close to the diagonal of *ρ* = 1 , as expected. (D) The last 8 responses from all the networks were averaged to produce the steady-state responsiveness of the system.

Figure 7 depicts the dynamics for a dual site stimulation paradigm. Stimuli were delivered either to site 1 (frequent) or to site 2 (rare) every 2 s (*f*_stim_ = 0.5 s^−1^), and the ratio between the stimulation intervals of the two sites was set to be 4:1, which corresponds to *β* = 0.2. Panel A depicts the network's dynamics for both sites, while panels B, C and D display the state variable dynamics for site 1 and site 2 local excitatory populations and for the global inhibitory population respectively.

**Figure 7:**
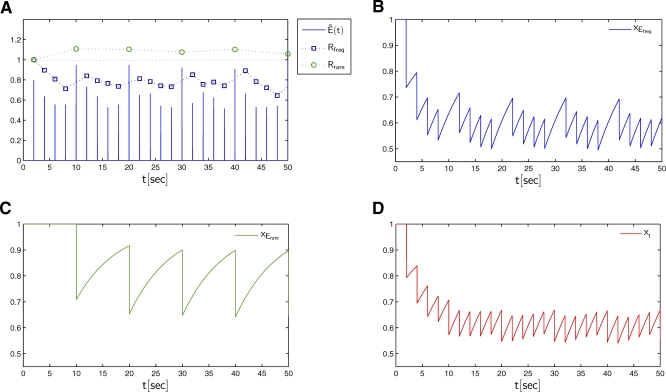
Dual Site Stimulation Dynamics in Simulated Networks (A) Network response to rare (circles) and frequent (squares) stimulation. Stimulation ratio was 4:1 (*β* = 0.2) at 0.5 s^−1^. (B) The average excitatory synaptic availability at the frequent site. (C) The average excitatory synaptic availability at the rare site. (D) The average synaptic availability of the global inhibitory population.

Performing dual-site stimulation on different networks with different values of *f*_stim_ and *β* allowed us to measure the selectivity and amplification variables, as defined in Equations 13 and 14 (Figure 8). The fixed point loci, as predicted in Figure 2, were illustrated by computing the average state variables.

**Figure 8:**
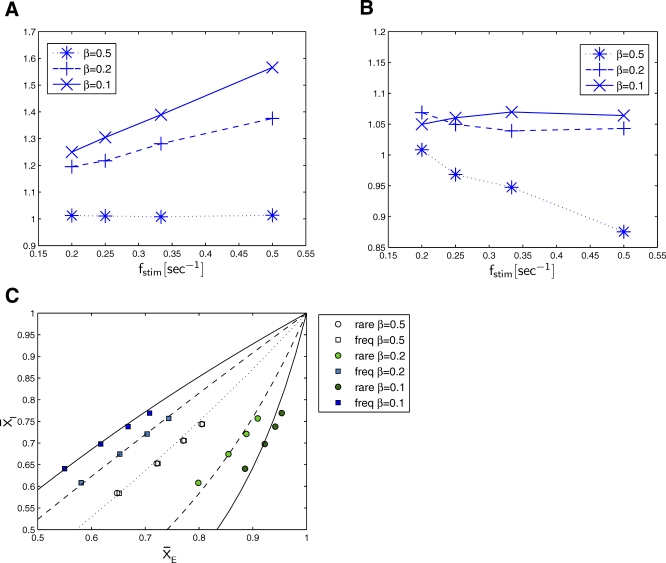
Selective Adaptation in Simulated Networks Results are averaged from 10 different random networks. (A,B) Dependence of selectivity (as defined in Equation 13) and amplification (as defined in Equation 14) on stimulation frequencies and relative intensity. For frequent-rare ratios greater than 1, selectivity increases with stimulation frequency. However, amplifcation (increased sensitivity to the rare) is pronounced only for high ratios. Both results agree with the theoretical predictions presented in Figure 4. (C) Dependence of the fixed point loci for the frequent (squares) and rare (circles) sites on frequencies and ratios. For a 1:1 ratio (*β* = 0.5), both sites are identically stimulated and so converge to the same fixed point in the phase plane. As *β* is decreased, *ρ_rare_* decreases while *ρ_freq_* increases. The curves were fitted according to Equation 9, values of *ρ* for each curve were found by trial and error: for *β* = 0.1, *ρ_rare_* = 0.2, and *ρ_freq_* = 1.45 (solid lines); for *β* = 0.2, *ρ_rare_* = 0.35 and *ρ_freq_* = 1.1 (dashed lines); for *β* = 0.5, *ρ_rare_* = *ρ_freq_*= 0.75 (dotted line). Using the identity *ρ*_0_ = *ρ_rare_* + *ρ_freq_* we can estimate that for this system *ρ*_0_ ≈ 1.5.

### Experimental Results

To finally corroborate our model, we performed adaptation experiments in networks of cortical neurons cultured in vitro. The results are presented in Figure 9. Panel A shows frequency dependent adaptation results from 19 networks, stimulated at a single site at various frequencies. Each marker represents the steady-state responsiveness (i.e., the total number of spikes observed in the network within 150 ms after a stimulus), normalized to the initial response of each network. A marked decline in responsiveness appears for rates larger than 0.1 s^−1^, while the networks fail to respond steadily when stimulated above 0.5 s^−1^.

**Figure 9:**
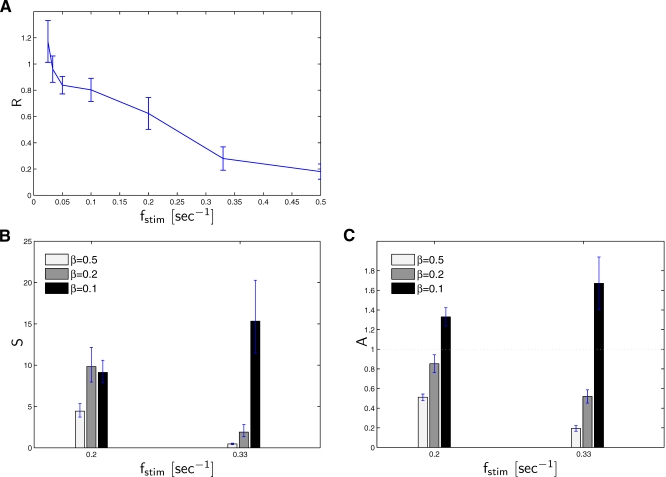
Experimental Results from Neural Cultures Developing In Vitro (A) Frequency dependent adaptation. A total of 19 networks were stimulated at different frequencies (0.02–0.5 s^−1^). At each frequency the networks were stimulated for 900 s, long enough to reach steady state. Responsiveness is defined as the mean number of spikes detected in a network within 150 ms after a stimulus. Steady-state responsiveness was computed by averaging over the last 6 responses following repetitive stimulation at a given frequency *f*_stim_. The result was normalized to the networks' initial responsiveness to produce *R* as defined in Equation 3. Error bars mark ±SEM. (B, C) Dual site stimulation. Stimuli were applied at two stimulation rates (once every 5 s and once every 3 s) and in three patterns: 1:4, 1:9, and 1:1 (sites A and B, respectively; i.e., *β* = 0.2, 0.1, and 0.5, respectively) in a paradigm similar to that described in [14] (total of 6 different stimulation epochs). Responsiveness is defined as the total number of spikes in the network within 150 ms after a stimulus. The network was allowed 15 min of recovery between epochs. (B) Dependence of selectivity (as defined in Equation 13). (C) Amplification (as defined in Equation 14) on stimulation frequencies and ratios. Steady-state responsiveness was computed by averaging over all responses to a site in the last half of each epoch. These were normalized to the un-adapted response, defined as the maximal response in the first 30 s of each epoch (this definition was used to overcome rapid transients in the initial response). Error bars in (B) mark S_min_ and S_max_ (see Materials and Methods, Equation 28). Error bars in (C) mark ±SEM (normalized to the un-adapted response).

Next, a network was stimulated at two sites (∼1 mm apart) following the paradigm in [14], using different stimulation rates (1/3 s^−1^ and 1/5 s^−1^) and ratios (1:1, 1:4 and 1:9, *β* = 0.5, 0.2 and 0.1, respectively). Thus we obtained 6 measurements from which we computed selectivity (Figure 9B) and amplification (Figure 9C) for each scenario according to Equations 13 and 14. Comparing these results with those presented in Figure 4 and Figure 8 we can conclude that for high stimulation ratios (*β* = 0.1), both selectivity and amplification exhibit the expected frequency dependence. At lower ratios, however, selectivity decreases as frequency is increased, which means that the site chosen for rare stimulation undergoes more pronounced depression. In terms of our model, this means that when stimulation frequencies are identical *β* > 0.5 for this choice of stimulation sites.

### A Network Model with Tonic Activity

So far we have applied our approach to systems with transient, phasic response to external stimulation and a low level of tonic activity. To demonstrate our approach in another, qualitatively different system, we now consider a Wilson-Cowan [19] type system, consisting of excitatory and inhibitory components,

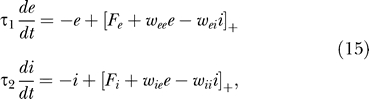
where *τ*_1_ and *τ*_2_ are the characteristic time constants, *e* and *i* the activities and *F_e_* and *F_i_* the feedforward stimulation intensities of the excitatory and inhibitory components. The components' interactions are mediated by the non-negative synaptic weights *w_ij_*. The “+” subscripts indicate that negative terms are clipped to zero. Such schematic systems have been used in many theoretical studies (see, for example, [20] section 7.5).

To apply our model for selective adaptation to this system, we assume that the external drive to the inhibitory population is negligible (compared with the excitatory and recurrent drive to this population), namely *F_i_* ≅ 0. The system with this additional assumption is illustrated in Figure 10.

**Figure 10:**
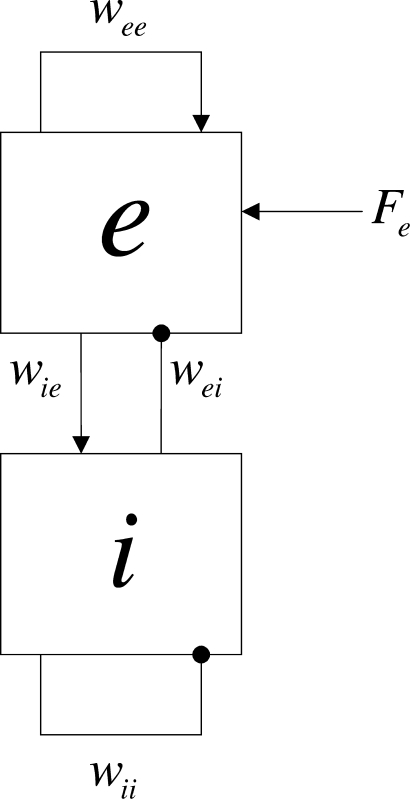
Model for Network with Tonic Activity The model consists of two coupled populations, excitatory and inhibitory. We assume the inhibitory population is not driven directly by external stimulation. Each population responds via a threshold linear response function, as described in Equation 15.

Now let us assume that the synaptic efficacies are subject to depression processes,

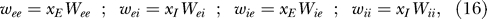
where *W_ij_* are the maximal synaptic efficacies and *x_E_* and *x_I_* are the relative resource availabilities, which follow the dynamics of Equation 2. We also assume that the neural activity time constants *τ*_1_ and *τ*_2_ are much shorter than the synaptic availability time constants *τ*_E_ and *τ*_I_, so that activities reach their steady-state values instantaneously. Defining

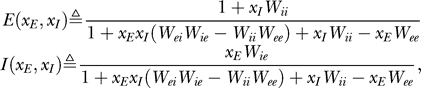



the steady-state solution of Equation 15 is given by

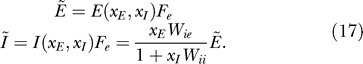



As can be seen the steady-state activities have a multiplicative dependency on external stimulation intensity, as required by our model. Local stability of the solution given in Equation 17, ensuring convergence to a fixed point, is guaranteed for all *x_E_*, *x_I_* if *W_ee_* < 1 (see Materials and Methods). Unstable solutions, where the system develops oscillatory activity, will not be discussed here.

Next we check the system's compliance with the assumptions in Equations 4–6. Assumption 4, namely the explicit dependence of the inhibitory activity on the excitatory activity is readily seen in Equation 17. Note that this dependence differs from the proportionality assumed in Equation 8, in that the ratio *I/E* (i.e., the function *g*(*x_E_*, *x_I_*) in Equation 4) is no longer constant. This difference between the two systems changes the phase plane structure but not the qualitative behavior, as discussed below. Differentiating with respect to the excitatory resource availability we have

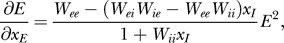
and therefore the monotonicity condition in Equation 5 is satisfied in this system when

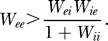



Differentiating with respect to the inhibitory resource availability we have

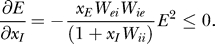



And therefore the condition in Equation 6 is indeed satisfied for all *x_E_*, *x_I_*.

The fixed points of the system are located on curves in the phase plane,

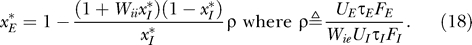



*F_i_* is the stimulation intensity affecting the inhibitory population indirectly through the excitatory drive to it, and may differ from *F*_e_ when two stimulation sources are present, as explained below. The curves, presented for different values of *ρ* in Figure 11, differ from those in the transient response system due to the different dependence described above. However, as 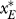 and 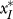 approach unity this difference vanishes. Moreover, in what follows we demonstrate that the qualitative nature of selective adaptation is maintained in this system. Equation 18 can be simplified assuming *W_ii_* ≪ 1, in which case we get

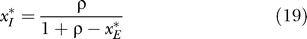



**Figure 11:**
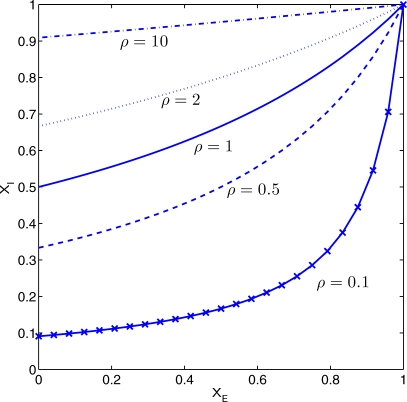
Fixed Point Loci for Networks with Tonic Activity Geometrical loci of fixed points for the system with activity functions described in Equation 17. The locus depends on the parameter ratio *ρ* following Equation 18. Note the difference between the phase plane structure in this system and the phase plane presented in Figure 2. The difference is due to the different relationships between inhibitory and excitatory activity (compare Equations 17 and 8). (*W_ii_* = 0.3).

with *ρ* defined as in Equation 18. In fact, the curves in Equations 18 and 19 are similar even when *W_ii_* approaches unity.

### Selective Adaptation in Networks with Tonic Activity

We now consider the two site stimulation scenario for this system. The model for this system is presented in Figure 12, resembling our conceptual model in Figure 1. The inhibitory population is global and therefore is driven by both excitatory populations. In the tonic system, however, instead of rare and frequent stimulation sites we have “strong” and “weak” sites. The responses at these two sites are

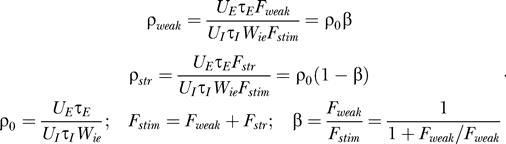



**Figure 12:**
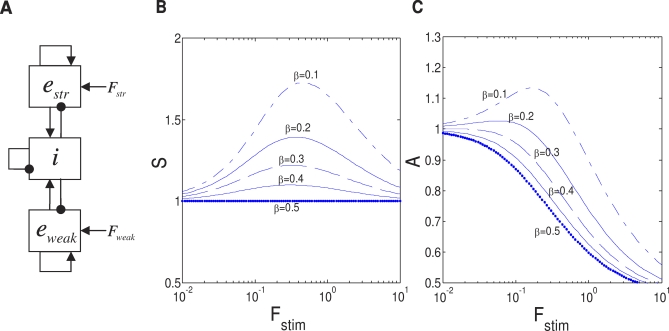
Selective Adaptation in Networks with Tonic Activity (A) schematic diagram of the dual site stimulation scheme. (B, C) Dependence of selectivity and amplification on total stimulation intensity *F*_stim_ (logarithmic scale) and relative intensity *β*. At low intensities response at both sites is high and behavior is similar to the one described for networks with transient responses (see Figure 4). At high intesities responsiveness is low and selectivity and amplification deteriorate. (*W_ee_* = 0.9; *W_ei_* = 0.75*; W_ie_* = 0.75*; W_ii_* = 0.3).

We define selectivity and amplification in this system using Equations 3, 13, and 14. The dependence of these measures on total and relative stimulation intensities, *F*_stim_ and *β*, is presented in Figure 12. The figures are sketched in logarithmic scale so two regions of behavior appear. At low intensity of stimulation (low *F*_stim_), where both sites are highly responsive, selectivity is monotonically increasing with input intensity. At high intensity of stimulation (high *F*_stim_) the system's responsiveness becomes very low and therefore selectivity and amplification decline. As mentioned above, such behavior was also observed outside the physiological bounds in the transiently responding network model (data not shown).

## Discussion

In this work we have formalized a model describing the various adaptation phenomena in neural populations. In many studies, adaptation was modeled in terms of cumulative effects of stimulation on either the exciting or the restoring resources of the system. These changes in resource availability lead to either a decrease or an increase in the system's responsiveness. When spatial segregation between the stimulation pathways is introduced, the effect of stimulation on the resources is local. Such a system will exhibit selective adaptation, the ability to adapt to one source while preserving its sensitivity to another. Far less trivial is the phenomenon we term “amplification,” which is an increased sensitivity to rare stimulation on the background of frequent stimulation. This phenomenon characterizes the MMN evoked potential and was also observed in vitro.

The ability of a network to perform selective adaptation has obvious functional implications. Applied as a filter in a sensory information pathway, for instance, this system can implement novelty detection, enhancing the propagation of rare sensory events while attenuating frequent events. Selective adaptation is one of the few phenomena observed in neural networks developing in vitro that has a well understood, non-trivial biological function. As such, it can serve as a benchmark to assess the functional complexity of such networks.

We developed a simple model describing the effect of external stimulation on each component of the system. This model was implemented on two types of neural systems, the first characterized by a transient response and the second by a tonic response. The model suggests that non-trivial adaptation phenomena can arise in a system with a relatively simple structure. The key requirement from the model is that the restoring resource (i.e., the inhibitory component) is affected globally from all stimulation pathways.

To test our model, we observed the dependence of both the system's selectivity and amplification on stimulation intensity, and on the ratio between intensities at two stimulation sites. For the transient responses network, the model predicts that within the physiological bounds (i) selectivity increases as stimulation frequency is increased, (ii) this sensitivity is more pronounced as the ratio between frequencies of rare and frequent stimuli increases and (iii) amplification is also frequency dependent, but can only occur at high ratios, i.e., when the stimulation rates are very different. These predictions were compared with computer simulations and with data from recordings in neural cultures developing in vitro.

In addition to predicting novel phenomena, our mathematical formalization provides further insight into adaptation processes. One clear benefit of a mathematical formalization involves the reduction of several variables to a single variable, which suffices to fully describe the behavior of interest, akin to an order parameter in physics. For instance, it has been reported in several studies that synaptic depression parameters differ between excitatory and inhibitory neurons [21]. This asymmetry in adaptive behavior formed the basis of a recent theoretical study [22]. According to our model, all the stimuli-independent physiological constants are reduced to a single ratio ρ_0_ that determines the system's steady-state behavior. Finally, a distinct benefit of our formal description is the ability it provides to generalize from our specific experimental system to new systems, sharing its basic characteristics, such as the tonic activity system presented in this paper.

As mentioned above, one of our main assumptions is that the inhibitory population serves as a global component in the system. We implemented this assumption in our model by separating the excitatory population into two segregated networks, both connected to a common inhibitory population. This was also the design we used for the leaky integrate-and-fire neural network simulation. Admittedly, this structure is a limiting case for our assumptions on the sub-networks' topologies, as one might expect that a more realistic network will include some interactions between the two excitatory sites and some degree of locality in the inhibitory one. In such a network the stimulation sites are not as distinct and therefore are affected more similarly by the stimuli. In other words, *β* will be closer to 0.5, where the response to the two inputs is identical. In that sense, *β*, which in our “extremist” model relates only to the difference between the inputs, may also represent the differences in component structure.

Another key assumption used in the development of our model is that the system has separable time scales; a fast time scale governing short term transients (e.g., membrane voltage, fast depression and facilitation) and a slow time scale (e.g., slow synaptic depression). This separation enabled us to extract the “slow evolution” of the system's state variables, either by using averaging methods (as was done in the transiently responding network example) or by assuming instantaneous response (as was done in the tonicly firing network example). One must note that all information conveyed in the fast dynamics of the system (e.g., latency, pattern of firing, etc.) is ignored using this technique.

In this work we have dealt with systems receiving input from one or two sources. An appealing possibility for future work is to broaden the model to systems receiving input from a continuum of input sources. For instance, an auditory cortical column responds to a range of stimulation frequencies. We hypothesize that the correlate of selective adaptation in such systems, the so-called “repulsive shifts” in their tuning curves (e.g., [8,22]), may arise from differences in the connectivity of the inhibitory and excitatory populations composing these systems. A model of such systems can also include more realistic connectivity schemes, as discussed above.

It is noteworthy that an essential component of our model is the globality of the inhibitory network, owing mostly to the electrical interconnections of this population via gap junctions (see [16–18]). This offers a compelling role to the abundance of connexins in the inhibitory neurons of the neocortex (e.g., [23]). A straightforward prediction of this hypothesis, then, is the abolishment of amplification in the absence of functional gap-junctions. An experiment verifying this prediction is yet to be performed.

To summarize, in this contribution we have developed a methodology that enabled us to reduce complex, heterogeneous networks to a minimal set of state equations. This procedure was implemented in order to investigate adaptation processes, which are vital features of biological systems in general, and neural networks in particular.

## Materials and Methods

### Derivation of state equations for transiently responding neural networks.

We consider a network composed of excitatory and inhibitory neurons with a very low level of intrinsic (spontaneous) activity. The network responds to a short (less than 1 ms) external stimulus in a reverberating episode of increased activity (several tens of ms), which we will refer to as the transient response (TR, sometimes referred to as “Network Spikes” or “Bursts”; see [24–27]). Unlike a stereotypical action potential of single neurons, however, this response is graded, namely the magnitude of each response varies and depends on the current state of the system. When stimulated repeatedly, the system's response was shown to decrease monotonically with stimulation frequency [14,15,28]. It was suggested that this decrease depends heavily on a decline in synaptic availability [29]. Based on these observations, we formulate below a model in which synaptic resource availabilities play the role of state variables.

We consider two populations of synapses, where each synapse (inhibitory or excitatory) is subject to short term plasticity modeled by a differential equation [30]

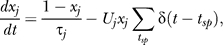
where *x_j_* represents the relative availability of release-ready synaptic vesicles, (*t_sp_*} denotes the times of firing by the pre-synaptic neuron; synaptic parameters are τ*_j_* (recovery time constant) and *U_j_* (the fraction of vesicles released for each pre-synaptic action potential). Between firing times *t_n_* and *t_n_*_+1_, the synapse recovers exponentially towards 1,

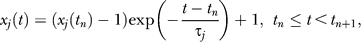
while upon the arrival of a pre-synaptic spike at time *t_n_*, the synapse utilizes a fraction of its resource (i.e., releases a fraction of the available vesicles)

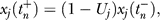
where 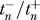 denote the time immediately before/after the *n*-th spike. We note that this equation guarantees that *x_j_* remains in the range 0–1.

Typically in mean field analysis of neural ensembles, one assumes that individual neurons fire as non-homogeneous Poisson processes and are sparsely connected to each other. Since the present analysis focuses on synaptic ensembles rather than neural ensembles, and since all synapses sharing the same pre-synaptic neuron receive the same input, these assumptions do not hold. However, if we assume that the total number of synapses in the network is much larger than the number of synapses per neuron and that connectivity is approximately uniform, we can neglect these inter-synaptic correlations (similarly to [30]). We can then average over the ensemble of synapses to get the mean population behavior

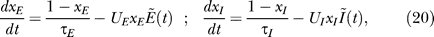
where *x_E_*, *x_I_* are the average available synaptic resources for the excitatory and inhibitory populations, respectively. The average activity rates in these populations, 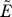 and 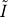 , depend on the available synaptic resources but vary with time as a result of “fast” processes and external stimulation.

Now let us assume that each population (excitatory and inhibitory) is stimulated periodically with period *T_E_* or *T_I_* and responds to each stimulus with a short transient response (TR). Since we are interested in the cumulative effect of stimulation on the network, we average each equation over a single period of stimulation

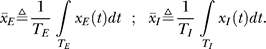



We can take the state variables to be relatively constant during a single response, which simplifies the integration

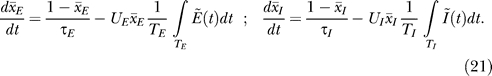



When the duration of the transient response *T_TR_* is much shorter than the stimulation periods *T_E_* and *T_I_*, the integration is independent of these periods. Furthermore, the outcome of this integration depends only on the synaptic availabilities at the stimulus onset. We define the populations' integrated “activity functions” as

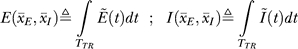



Substituting these definitions in Equation 21 we obtain the averaged state equations

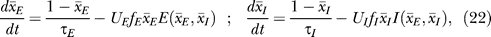
where *f*_E_=1/*T*_E_ and *f*_I_=1/*T*_I_ are the stimulation rates.

### Fixed point location.

The fixed points of the averaged system (Equation 22) are located where both derivatives vanish, namely

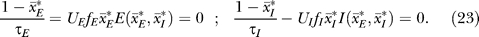



Dividing the first equation by the second one we get

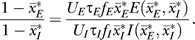



We can reasonably assume that

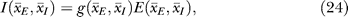



where *g* is some function of the resource availabilities (this relationship is plausible when the inhibitory population is not driven by external sources, e.g., in the tonic system discussed above).

Let us consider the simple case where 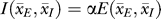 with *α* > 0. The fixed point equation under this assumption reduces to

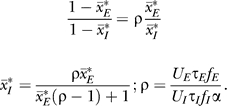



Therefore, the parameter ratio determines the geometric locus (curve) of the system's fixed points. The exact location of the fixed point on this curve is governed by the specific parameter values and activity functions *E, I*.

### Stability analysis.

The asymptotic stability of the system's fixed point is determined by the eigen values of the Jacobian matrix at these points

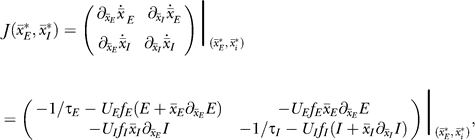
where ∂*_x_* denotes partial derivation with respect to *x*. Necessary and sufficient conditions for stability are (see [31], section 5.2):

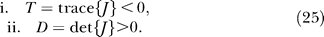



We consider the simple case of a symmetric system in which 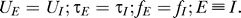


In this system, *ρ* = 1 so that the fixed points are at 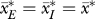 .

Computing the stability criteria in Equation 25 for this system yields

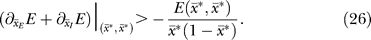



We note that the activity function's directional derivative along the diagonal 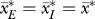 (i.e., the derivative along the fixed point loci) is

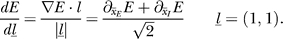



Thus, the stability criterion in Equation 26 can be rewritten

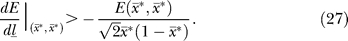



Specifically, if the system's activity depends more strongly on the availability of excitation than on that of inhibition, then its activity increases along the diagonal,

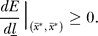



In this case response is non-increasing with stimulation frequency, Equation 27 is always satisfied and the fixed point is stable. This is easily explained intuitively as a negative feedback arising between the state variables and the activity.

### Stability analysis of the tonic system.

Since we assume the neural activity time constants, *τ*_1_ and *τ*_2_, are much shorter than the synaptic availability time constants *τ_E_* and *τ_I_*, we can analyze the stability of Equation 15 assuming *x_E_* and *x_I_* are fixed, since they change little, while *e* and *i* change. The Jacobian matrix derived from the system in Equation 15 is

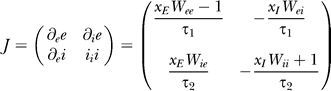
(see stability analysis of 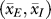 , above). The trace of this matrix is negative when

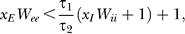
which is fulfilled for all (*x_E_*, *x_I_*) when 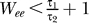 . The determinant is positive under the condition

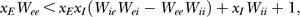
which is fulfilled for all (*x_E_*, *x_I_*) when *W_ee_* < 1. We conclude that both stability conditions are met when *W_ee_* < 1.

### Simulation.

The simulation included a network of leaky integrate and fire equations

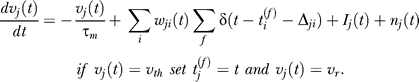



Each synapse is characterized by its efficacy *w_ji_* and delay Δ*_ji_*. Two synaptic depression processes were modeled, based on [21] and [32]

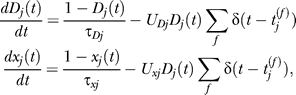
where *D_j_* and *x_j_* are the fast and slow synaptic depression variables, respectively. The synaptic efficacy of each neuron is determined by the equation

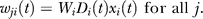



In the single site stimulation paradigm, a network of 100 excitatory neurons and 40 inhibitory neurons was simulated, each driving 12 synapses (connectivity was uniformly random). In the dual site stimulation paradigm, 200 excitatory neurons were separated into two segregated populations, both connected to the “global” inhibitory population randomly.

Stimuli were applied periodically by injecting a current pulse randomly into 10% of the stimulated neural populations

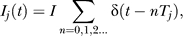
where *T_j_* is the stimulation period.

Each neuron is also subject to random white Gaussian noise

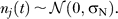



Table 1 summarizes the values used for each parameter mentioned in the above description. These were chosen so that the system's time-scales resemble those in [14]. In particular, the system is non-responsive to stimulation beyond 0.5 s^−1^, and adaptation is significant beyond 0.1 s^−1^.

**Table 1:**
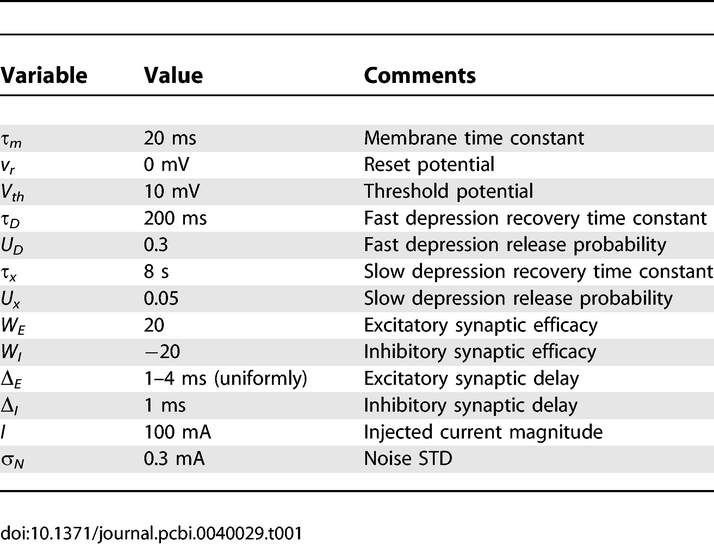
Summary of Simulation Parameter Values

### Cultured networks.

Cortical neurons were obtained from newborn rats within 24 h after birth, following standard procedures as described previously [25,33]. The cortical tissue was enzymatically digested and mechanically dissociated. The neurons were plated directly onto substrate-integrated multi-electrode array (MEA) dishes [34]. The cultures were bathed in MEM supplemented with heat inactivated horse serum (5%), glutamine (0.5 mM), glucose (20 mM), and gentamycin (10 μg/ml) and maintained in an atmosphere of 37 °C, 5% CO_2_ and 95% air in a tissue culture incubator as well as during the recording phases. Experiments were performed during the third week after plating, thus allowing functional and structural maturation of the neurons [33].

### Electrophysiology.

We used arrays of 60 Ti/Au/TiN electrodes, 30 μm in diameter and spaced 200 μm from each other (MultiChannelSystems, Reutlingen, Germany). The insulation layer (silicone nitride) was pretreated with poly-D-lysine. A commercial 60-channel amplifier (B-MEA-1060, MultiChannelSystems) with frequency limits of 1–5,000 s^−1^ and a gain of 1,024*×* was used. The B-MEA-1060 was connected to MCPPlus variable gain filter amplifiers (Alpha-Omega, Nazareth, Israel) for further amplification. Stimulation through the MEA was performed using a dedicated eight-channel stimulus generator (MultiChannelSystems). Data were digitized using two, parallel 5200a/526 analog-to-digital boards (Microstar Laboratories). Each channel was sampled at a frequency of 24 kilosample/s and prepared for analysis using the AlphaMap interface (Alpha-Omega). Thresholds (8X rms units, typically in the range of 10–20 μV) were defined separately for each of the recording channels before the beginning of the experiment. Before each experiment and between stimulation epochs, the networks were monitored for at least 15 min to ensure stability of the activity and to allow recovery.

Selectivity error bounds were defined as

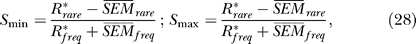


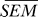
 is the standard error of mean responsiveness, normalized to the un-adapted response.
